# Surfactant proteins A and D nucleotide variants: association with retinal vascular disease

**DOI:** 10.1038/s41390-025-04435-w

**Published:** 2025-10-16

**Authors:** Kelsey Brass Allen, Dustin Rousselle, Christopher E. Aston, Keishla Colón Montañez, Patricia Silveyra, Wen Chen, Jeffrey Eckert, Raymond Michael Siatkowski, Peter F. Vitiello, Faizah N. Bhatti

**Affiliations:** 1 Section of Neonatal-Perinatal Medicine, Department of Pediatrics, University of Oklahoma Health College of Medicine, Oklahoma City, OK USA; 2 Department of Environmental and Occupational Health, Indiana University School of Public Health-Bloomington, Bloomington, IN USA; 3 Biomedical and Behavioral Methodology Core, Department of Pediatrics, University of Oklahoma Health, College of Medicine, Oklahoma City, OK USA; 4 Department of Ophthalmology and Dean McGee Eye Institute, University of Oklahoma Health College of Medicine, Oklahoma City, OK USA; 5 Department of Cell Biology, University of Oklahoma Health College of Medicine, Oklahoma City, OK USA

## Abstract

**Background:**

Retinopathy of prematurity (ROP) is associated with systemic inflammation. Surfactant proteins A and D (SP-A and SP-D) play an immunomodulatory role. We previously reported the impact of SP-A on retinal angiogenesis. This study investigates SP-A and SP-D single-nucleotide polymorphisms (SNPs) with risk of ROP.

**Methods:**

Subjects were infants with gestational age (GA) of <32 weeks and/or birth weight <1500 grams. DNA from blood was used to genotype the SNPs. Statistical analysis used logistic regression for the association of ROP with genetic and clinical factors, including bronchopulmonary dysplasia (BPD), GA, and oxygen exposure.

**Results:**

A total of 59 infants were enrolled. In the whole cohort, the SFTPA1 SNP rs1059057 ‘G’ allele was associated with increasing odds of ROP when controlling for GA and oxygen. In both the whole cohort and in BPD, the SFTPA2 SNP rs1965707 ‘T’ allele was associated with increasing odds of ROP risk when controlling for GA and oxygen. Furthermore, there was an interaction effect where the protective effect of GA in the presence of the wildtype (C/C) was diminished in the presence of the ‘T’ allele.

**Conclusions:**

The study identifies novel associations between surfactant protein gene SNPs and ROP risk that may impact protein structure, function in the retinal vasculature.

**Impact:**

- Retinopathy of Prematurity (ROP) stems from disrupted angiogenesis.
- Surfactant protein A (SP-A) impacts retinal vascular disease, but associations between genetic polymorphisms of surfactant proteins (SPs) and ROP are unknown.
- We report novel surfactant protein polymorphisms and ROP. The risk of ROP modeled by controlling for gestational age (GA) and oxygen suggests novel direct effects on endothelial function and angiogenesis.
- The resultant amino acid substitutions were mapped to predict translational protein modifications.
- As SNPs alter amino acid structure, protein folding, and functionality, our results provide critical mechanistic clues for vascular diseases of prematurity. Mapping of genetic signatures enables earlier detection of ROP.

## Background

Retinopathy of prematurity (ROP) is the leading cause of acquired childhood blindness worldwide, with the incidence almost doubling from 2003 to 2019.^1^ In the United States and Canada, an estimated 40% of infants born prematurely develop some degree of ROP, and approximately 4-6% infants have ROP severe enough to warrant treatment. However, milder forms of ROP are associated with lifelong visual deficits.^2–4^ Worldwide, 4% of preterm infants with ROP become legally blind^3^ every year. Lower birth weight and gestational age, fluctuations in oxygen tension, poor growth, and inflammation are all significant risk factors in the development of ROP,^5,6^, but a single causal factor is lacking. Until recently, the larger body of work has focused on oxygen-dependent vascular growth factors. However, inflammation and dysregulation of developmental vascular signaling pathways are now under greater scrutiny. Current therapeutic modalities targeting oxygen-related factors are only administered *after* neovascular disease and visual deficits have developed, and these treatments may still result in residual lifelong visual and neurological deficits. Thus, it is imperative to improve our understanding of how disruptions in vascular signaling pathways during critical developmental windows may be targeted for prevention, which can aid in shifting programmatic paradigms towards earlier detection of ROP.

The surfactant proteins A and D (SP-A and SP-D) are C-type lectins that regulate immunomodulatory pathways in preterm infants,^7^ acute respiratory infections,^8–10^, and in pulmonary carcinoma.^11^ SP-A is known to be deficient after birth in preterm infants, with pulmonary levels rising over the first 5-6 weeks of life^12^, both physiologically as well as in response to inflammation.^13–20^ Similar to reports in humans, animal studies have shown a similar trend with increasing pulmonary and retinal SP-A levels after birth.^21,22^ Classically, dysregulation and/or genetic modifications of surfactant proteins have been linked to respiratory distress syndrome (RDS) and bronchopulmonary dysplasia (BPD) in preterm infants; however, their scope and function are being increasingly recognized in other organs and systems. Studies from our laboratory have shown that surfactant proteins are expressed in multiple retinal cell types, are in close proximity to retinal vascular structures, and that SP-A is associated with a pro-angiogenic phenotype (angiogenesis)^7,22,23^ during both early vascular arrest and secondary neovascular disease.

**Fig. 1:**
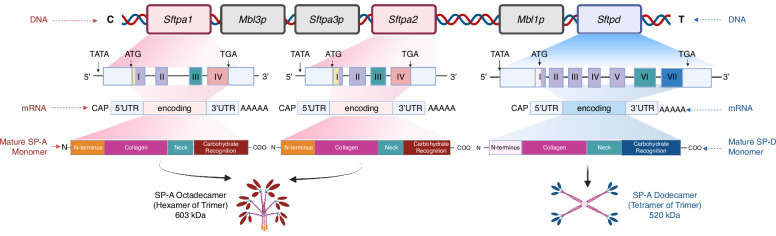
Genomic, translational, and transcriptional depictions of SFTPA1, SFTPA2, and SFTPD with mature protein structure. Each monomer has four distinct regions: N-terminal, a collagen-like region, neck and carbohydrate recognition domain. Three gene products form a trimer for SP-A protein. The final conformation is a hexadecameric (18 gene products) folded into a bouquet of tulips formation. Four gene products of SFTPD form the SP-D protein. UTR untranslated region, SFTPA surfactant protein A, SFTPD surfactant protein D, MBL mannose binding lectin, mRNA messenger RNA. *Created in BioRender. Bhatti, F. (2025) https://BioRender.com/tblrmjc.

**Fig. 2:**
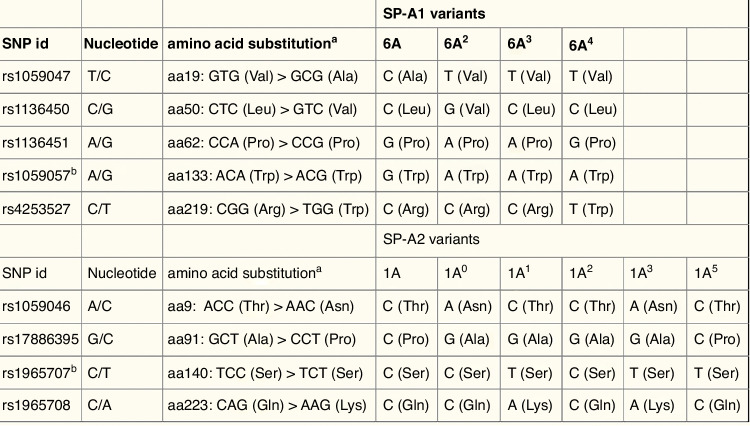
Variants of SFTPA1 and SFTPA2. The individual SNPs in the coding regions of SFTPA1 and 2 are denoted with resultant amino acid substitutions. The location of each SNP is shown in relation to the allelic variants (haplotypes) for each protein. These SNPs were chosen as they are the most frequently reported in the literature and in association with the variants shown. THR threonine, ASN asparagine, ALA alanine, PRO proline, SER serine, GLN glutamine, LYS lysine, VAL valine, LEU leucine, TRP tryptophan, MET methionine. Figure adapted from Silveyra et al.^32^.

In primates, the mature SP-A protein is encoded by two duplicated genes, SFTPA1 and SFTPA2,^7^, with six common polymorphisms located in coding regions, and several reported haplotypes.^24^ SFTPD has two predominant polymorphisms, Met11Thr and Ala160Thr, each having a minor allele frequency exceeding 20%.^25^ All three genes are located on chromosome 10; the genes, transcripts, and translated proteins with mature confirmation are depicted in Fig. 1 (adapted from Vieira et al. 2017). Previous studies showed that single-nucleotide polymorphisms (SNPs) in the surfactant protein genes are associated with the development of both early (RDS) and late (BPD) chronic lung disease.^26–34^ These SNPs may confer protection or render the condition more susceptible, depending on the coding region affected as well as the ethnic group/race being studied. Both the SFTPA1/SFTPA2 haplotype 6A^2^/1A^0^ and the SFTPD/SFTPA2 haplotype DA160(A)/SFTPA2(1A^1^) have been associated with protection from severe lung disease^27,30,33^ even after controlling for various environmental factors. The allelic variants with the respective location of SNPs and amino acid substitutions are denoted in Fig. 2 (adapted from Silveyra et al.^7,32^).

Studies from our lab have focused on rodent models of ROP to gain mechanistic insight into SP-A-driven vascular signaling pathways. However, rodents differ from primates as they express only one SP-A gene, SFTPA1. Therefore, it is critical to define these polymorphisms and their impact on the retinal vascular phenotype in human infants based on genetic specificity. Studies examining SNP associations must be evaluated with extreme caution in extra-pulmonary systems, as the immunomodulatory dysregulation in the pulmonary system and severity of lung disease may drive and compound the magnitude and manifestations of systemic co-morbidities in preterm infants.

As no previous reports have assessed surfactant protein polymorphisms in either ROP or human eye disease, we designed this study to interrogate the hypothesis that the SFTPA1/ SFTPA2 haplotype 6A^2^/1A^0^ decreases the risk of severe ROP (Stage III or greater/Zone II or less) or ROP requiring treatment. Identifying significant SNPs and variants is critical in improving genetic testing to predict severe ROP earlier in at-risk infants. Elucidation of genetic variants of surfactant proteins is also critical for understanding the mechanisms by which they impact endothelial cell function in early human development and will guide targets for personalized medicine. Here we report on the associations of SP-A1, SP-A2, and SP-D SNP variants and haplotypes with the risk of ROP after accounting for the effects of GA at birth and duration of treatment with oxygen in both preterm infants, regardless of BPD status, and in those with BPD.

## Design/Methods

### Study design

This was a pilot, prospective, case-control observational study at the Oklahoma Children’s Hospital, University of Oklahoma Health Sciences Center (OUHSC), conducted after Institutional Review Board (IRB) approval (IRB #6568) in accordance with the institutional ethical standards, between 2014–2022. As a pilot study, it was initially planned to enroll 100 infants with ROP and 100 infants with no ROP. After enrollment of 50 infants in total, an interim analysis found several significant SNP associations. This was followed by enrollment of 10 additional subjects to ensure the rigor of the analysis.

Infants in the NICU with the following eligibility criteria were identified: birth at less than 32 weeks’ post-menstrual age (PMA), GA and/or birth weight less than 1500 grams, and no prenatal diagnosis of any major congenital anomalies (chromosomal/genetic disorders). Informed consent was obtained from the parents or legal guardians of the infants at any time between birth and discharge from the NICU. In addition, a second group of ten eligible subjects was identified from the IRB-approved (IRB #14187) Oklahoma Preterm Infant Biorepository (HEROES) at OUHSC protocol from 2022–2023, with samples and clinical data collected under the Biorepository protocol. All enrolled infants were prospectively followed until the time of discharge from the NICU. The determination of ROP status (present or absent) and staging of disease was made based on routine screening exams performed by our OUHSC pediatric ophthalmologist (RMS) at the schedule recommended by the American Academy of Ophthalmology/ American Academy of Pediatrics.

Clinical data for all enrolled infants were collected and stored in a secure REDCap database maintained by the Biomedical Research Core at OUHSC, with only de-identified data used in the analysis. Data included GA at birth, birth weight, sex, ethnicity/race, total days on supplemental oxygen, and presence or absence of BPD. The definition of BPD was based on the 2018 National Institute of Health^35^ criteria, rather than the Jensen criteria,^36^ as the role of oxygen exposure as a risk factor for ROP was being considered in this study. Oxygen exposure, or duration of oxygen treatment, was measured using total days of oxygen exposure (DO_2_) and defined as the total number of days having *both* > 2 L flow *and* > 21% FiO2. Associations of risk of ROP were analyzed first in the whole subject cohort and then in the BPD cohort that included only those infants with BPD. This allowed for stricter control of the effects of lung disease and oxygen exposure, and to differentiate it from the independent risk conferred by the SNPs and/or haplotypes with ROP.

Clinical data related to ROP were derived from screening retinal exams performed by our Pediatric Ophthalmologist at Oklahoma Children’s Hospital and included the presence or absence of ROP, and the highest/most severe stage of ROP occurring during the NICU hospitalization.

### Collection of biological samples for DNA

After enrollment, scavenged blood samples from routine blood testing from the laboratory or cheek swabs were collected for each enrolled subject for purposes of isolation of DNA.

### Genotyping of Variants

After sample collection, DNA was extracted using the QIAamp DNA Mini Kit according to the manufacturer’s instructions and quantified on a NanoDrop 2000 spectrophotometer. Once pooled, the samples were shipped on dry ice to the Silveyra Lab (PS)^32^ at Indiana University, for polymerase chain reaction (PCR)-based genotyping of the variants of interest. For each SNP one of four validated methods were used for determining the SNP genotypes:

**Method 1** used TaqMan allelic discrimination, essays with purified DNA. DNA samples were first diluted to 25 ng/µL and underwent qPCR with TaqMan genotyping master mix (Cat #4371355) and TaqMan assays specific to each SNP.

**Method 2** used gene-specific nested PCR in combination with TaqMan allelic discrimination assays.

**Method 3** used restriction fragment length polymorphism (in RFLP) analysis after gene-specific and amino acid-specific PCR (modified from DiAngelo et al. 1999).^25^

**Method 4** used sequencing after gene-specific and amino acid-specific PCR.

These methods are described in full detail in the **Supplementary Data-Methods**. The method used for each particular SNP was selected a priori based on validated methods used in prior studies and is indicated in Supplementary Data-Methods SM1. After each individual SNP was confirmed, the STFTP allelic variant for the SP-A related genes was assigned using the designation depicted in Fig. 2.^32^

### Statistical analysis

Group statistics are expressed as mean ± standard deviation (SD) for continuous measures or as count (percentage) for categorical measures. Logistic regression within a generalized linear model framework was used to assess the association of risk of ROP with continuous (GA, DO_2_, gene in an allelic dosage model) and/or categorical (gene in dominant or recessive models) factors. Genetic factors were primarily entered as either an additive (allelic dose) model (where the mutant homozygote has twice the effect on risk as the heterozygote) or a dominant model (where the mutant homozygote and the heterozygote have the same effect on risk); however, the recessive model and the codominant model (heterozygote effect is free to take any value between those of the wildtype and mutant homozygotes) were also considered for specific hypotheses, such as determining the best inheritance pattern of effect on risk for a specific gene. Individual SNPs were considered both separately as genetic factors and combined as haplotypes. Preliminary analysis of the distribution of SNPs haplotypes was used to assign the wildtype allele as the most common allele in the sample of patients who were not affected by ROP.

Initial tests of ROP risk considered the effects of the separate genes SFTPA1/SFTPA2/SFTPD alone [linear model: ROP = gene]. This was followed by an “adjusted” analysis that included the effects of GA at birth and duration of oxygen treatment (DO_2_) in the linear model [ROP = gene + GA + DO_2_]. For each specific gene variant, an analysis of gene × GA interaction effect was done [ROP = gene + GA + DO_2_ + gene.GA] where indicated by analysis of GA effects in sub-groups of genotypes. Akaike’s Information Criterion (AIC) was used as an estimator of prediction error and thereby relative quality of statistical models for a given set of data.^37^ Statistical analyses were performed in R (version 4.2.2) with the gmodel package (version 2.18.1). The threshold for statistical significance was set at *p* < 0.05.

## Results

A total of 59 subjects were enrolled in the study. Demographic characteristics and baseline clinical status are presented in Table 1. Within the entire cohort of the 59 infants, 30 infants (51%) were classified as extremely preterm (<28 weeks of GA), and 23 (39%) had any stage of ROP, out of which four infants underwent treatment (6% of total subjects and 17% of those with ROP. In those infants with BPD (*n* = 42, 71% of the whole cohort), 21 (50%) had ROP, of which four infants underwent treatment. Of those with BPD, 28 infants (67%) were classified as extremely preterm.

**Table 1:** Demographic and Clinical Characteristics of Subjects

		All Infants (*n* = 59)		Infants with BPD (*n* = 42)	
		n	%	n	%
Sex	Female	34	58	24	57
	Male	25	42	18	43
Gestational Age	Extremely preterm (<28 weeks’ gestation)	30	51	28	67
	Very preterm (28–32 weeks’ gestation)	29	49	14	33
Race	American Indian or Alaskan Native	7	12	5	12
	Black or African American	10	17	7	17
	Hispanic or Latino	2	3	1	2
	Native Hawaiian or Other Pacific Islander	1	2	0	0
	White/Not Hispanic or Latino	39	66	29	69
BPD	Yes	42	71		
	No	17	29		
Days on Oxygen	Range 0–175 days				
	0	5	8	0	0
	1–20	10	17	0	0
	21–40	11	19	9	21
	41–60	5	8	5	12
	61–100	18	31	18	43
	>100	10	17	10	24
ROP	Yes	23	39	21	50
	No	36	61	21	50
ROP stage (*n* = 23)	Z2 S2	1	4	1	5
	Z2S1	8	35	7	33
	Z2S2	10	44	9	43
	Z2S2 pre plus	2	9	2	9
	Z2S3	1	4	1	5
	Z2S3 pre plus	1	4	1	5
ROP treatment (*n* = 23)	Yes	4	17	4	19
	No	19	83	17	81

Data are shown for the entire cohort of infants (*n* = 59), and for the subset of infants with BPD (*n* = 42). BPD is defined as reported in by the NICHD criteria.^35^ ROP is defined as any stage of ROP, and the frequency of stages is shown. ROP staging follows the classification as reported in ICROP 3rd edition^57^ and as diagnosed by the pediatric ophthalmologist performing the screening exams. Treatment comprised of laser and/or intravitreal bevacizumab injections as deemed appropriate by the ophthalmologist as standard of care. BPD=bronchopulmonary dysplasia; Z=zone; S=stage.

Exposure to oxygen and respiratory status can both variably impact vascular development and can confound results, as low birth weight infants are at higher risk of developing both lung disease and ROP. We designated two nested cohorts for analyses a priori- first by evaluating the association of SNPs and risk factors for ROP in *the entire infant cohort* (*n* = 59), and the second was to evaluate these SNPs for ROP association *only in infants with BPD* (*n* = 42).

### Analysis of complete subject cohort

There were no significant associations of ROP seen with sex or race/ethnicity. As expected, in single variate analysis, the odds of developing ROP were decreased with increasing GA (*p* value = 0.0019; OR = 0.57, 95%CI [0.41–0.80], Table 2) but increased with increasing number of DO_2_ (*p*-value = 0.0002; OR = 1.04, 95%CI [1.02–1.06], Table 2. Similarly in infants with BPD, the odds of developing ROP were decreased with increasing GA (p value = 0.003; OR = 0.48; 95%CI [0.29–0.78]) but increased with increasing number of DO_2_ (*p* value = 0.0021; OR_=_1.05; 95%CI [1.02–1.08]).

**Table 2:** The association of gestational age and duration of oxygen use on the risk of ROP

	All Infants (*N* = 59)			Infants with BPD (*N* = 42)		
Individual Variables	OR	95% CI	*p* value	OR	95% CI	*p* value
**Increasing GA (weeks)**	0.57	[0.41–0.80]	**0.0019**	0.48	[0.29–0.78]	**0.0031**
**Increasing days on O**_**2**_	1.04	[1.02–1.06]	**0.0002**	1.05	[1.02–1.08]	**0.0021**
**Multiple variable (GA + days on O**_**2**_**)**						
**Increasing GA (weeks)**	0.79	[0.52–1.20]	0.26	0.61	[0.34–1.07]	0.082
**Greater days on O**_**2**_	1.03	[1.01–1.05]	**0.011**	1.04	[1.01–1.07]	**0.019**

GA and DO_2_ are first fitted as continuous variables individually using logistic regression and then jointly as a multiple variable model for ROP risk (ROP = GA + DO_2_). The “*p* value” is from a Wald’s test and is significant if the OR is significantly different from OR = 1. Note that for Infants with BPD a Likelihood Ratio test (LR) for [ROP = DO_2_] vs [ROP = GA + DO_2_] has *p* = 0.042 while the LR for ROP = GA vs ROP = GA + DO_2_ has *p* = 0.0052, thus adding either GA or DO_2_ as the second variable to form the multiple variable model shows a significant improvement in goodness-of-fit of GA makes a significant improvement to goodness-of-fit per the LR. Pearson’s correlation for increasing GA and increasing DO2 is *ρ* = −0.56 [95%CI: −0.74, −0.31]. *p*-value ≤ 0.05 are significant and highlighted in bold font; GA Gestational Age, DO_2_ days on oxygen, OR Odds Ratio, CI Confidence Interval.

The frequencies of SNPs with representative nucleotide substitutions in all infants (whole cohort) with and without ROP are shown in Supplementary Table S1, together with the incidence of ROP within each genotype. Supplementary Table S2 shows the genotype and haplotype frequencies for SFTPA1 and SFTPA2 haplotypes in infants with and without ROP, as well as incidence of ROP in infants with the genotype.

#### Association of ROP risk with SNPs (with and without covariates)

In the whole cohort analysis, two SNPs in *SFTPA2* showed significant association with ROP in the *unadjusted models* (see Supplementary Table S3). SNP rs1965707 was significantly associated with ROP under both the Additive (allelic dosage) model (*p* = 0.028) and the Dominant model (*p* = 0.011). For this SNP, the homozygote C/C was most frequent in infants without ROP, defining this as the Wt/Wt. The incidence of ROP in the heterozygote C/T and homozygote T/T was about the same (59% and 50%, respectively). The second SNP, rs17886395, was significantly associated with ROP but only under the Dominant model (*p* = 0.026); it showed perhaps marginal significance (*p* = 0.082) under the Additive model. The homozygote G/G was most frequent in infants without ROP, defining this as the Wt/Wt. The incidence of ROP in the heterozygote G/C was 61% but only 33% in the homozygote C/C. Small sample size may be an issue in the latter, as there were only three G/G, however, this was an issue common to most of the μ/μ homozygotes. The inclusion of *GA and duration of oxygen treatment* in the analyses made little substantive change to these results, as shown in Supplemental Table 4, where the “*p* value” is from a Likelihood Ratio test whether adding the haplotype to the risk model for ROP offers a significant improvement in goodness-of-fit. Here, rs1965707 showed a significant association with risk for ROP under both Additive and Dominant models, and rs17886395 showed a significant association with risk for ROP under the Dominant model, although all of the SFTPA2 SNPs shifted towards marginal significance with the inclusion of these covariates. However, the SNP rs1059057 in SFTPA1 did reach significance with the inclusion of these covariates under both Additive and Dominant models (*p* = 0.047), although the absence of any infants with the G/G genotype (the μ/μ homozygote) makes the Additive and Dominant models’ equivalent. There was no suggestion of a significant association of this SNP without the covariates.

#### Association of ROP risk with haplotypes (with and without covariates)

Frequencies and incidence of ROP are shown for the SFTPA1 and SFTPA2 haplotypes in Supplemental Table 2. Wt for SFTPA1 is haplotype 6A^2^ and for SFTPA2 is haplotype 1A^0^ defined as the ‘Most Frequent Allele’ (or haplotype). SFTPA1 haplotypes were analyzed using additive and dominant risk models (Wt = 6A^2^), both with and without covariates, but none showed evidence of significant association with the odds of developing ROP. SFTPA2 haplotypes were similarly analyzed using additive and dominant risk models (Wt = 1A^0^) without covariates, but none showed evidence of a significant association of these haplotypes with the risk of ROP. However, when analyzed including covariates GA and duration of oxygen treatment in the analyses, both Additive (p = 0.068) and Dominant (p = 0.059), while not reaching significance, were perhaps marginally significant, as was perhaps alluded to by the shift of all the SFTPA2 SNPs towards marginal significance with the inclusion of these covariates noted earlier.

## Analysis of the BPD Cohort

Results are now presented for the association of ROP with SNPs and haplotypes *only in infants with BPD* (*n* = 42) to consider the variation in risk for ROP due to the presence/absence of pulmonary disease and to focus on the effects of the genetic variants in the presence of pulmonary disease. Infants with BPD represent 71% of the complete cohort, but 91% of the infants with ROP.

### Gestational age and days on oxygen

As seen in the entire cohort, there were no significant associations of risk of ROP with sex or race/ethnicity. As expected, the odds of developing ROP were decreased with increasing GA (*p*-value = 0.0031; OR = 0.48, 95%CI [0.29–0.78], Table 2) and increased with increasing number of DO_2_ (*p*-value = 0.0021; OR = 1.05, 95%CI [1.02–1.08], Table 2). While adjusting for GA, DO_2_ remains a significant predictor of the development of ROP in those with BPD, GA appears to lose its significance as a predictor when adjusting for DO_2_, according to the Wald tests shown in Table 2. However, a Likelihood Ratio test (LR) for [ROP = DO_2_] vs [ROP = GA + DO_2_] has a *p* = 0.042 while the LR for [ROP = GA] vs [ROP = GA + DO_2_] has *p* = 0.0052, showing that adding either GA or DO_2_ as the second variable to form the multiple variable model shows a significant improvement in goodness-of-fit per the LR. We chose to retain GA in the multiple variable models as lower GA is accepted as a known risk factor in the development of ROP.

### Association of individual SNPs with ROP

The analysis for the association of SNPs with risk for ROP in infants with BPD is shown without covariates in Table 3A and in Table 3B with the covariates GA and DO_2_. Similar to the whole cohort analysis above, *SFTPA2* SNP rs1965707 was significantly associated with ROP under both the Additive (Allelic dosage) model (*p* = 0.0036) and the Dominant model (p = 0.0069), while for the Recessive model, we noted that all T/T homozygotes were affected with ROP. Again, the presence of the T allele carried increased odds for ROP. For this SNP, the homozygote C/C was most frequent in infants without ROP, maintaining this as the Wt/Wt. The SNP rs1965707 shows a significant association with risk for ROP regardless of the inheritance pattern of risk: Additive (Allele dosage), Dominant, or Recessive. To determine whether one of these models is favored over the others, a series of Likelihood Ratio tests (LR) was performed. For a codominant model (the effect of C/T is free to vary between those of C/C and T/T) vs Additive (Allele dosage) model (the effect of C/T is half that of T/T since C/T has half as many T alleles) has *p* = 0.52, i.e., the codominant model shows no statistical improvement over additive so, by Occam’s razor, the simpler (Additive) model is chosen. A Likelihood Ratio test (LR) for a codominant model vs a Dominant model (the effect of C/T equals that of T/T) has *p* = 0.15, indicating no statistical improvement of Codominant over Dominant. Thus, by Occam’s razor, we choose the simpler (Dominant) model. Additive vs Dominant cannot be tested directly; however, the Akaike’s Information Criterion (AIC) for each model term can be compared, with the lowest AIC indicating the best model, as shown in Table 4. AIC deals with the trade-off between the goodness of fit of the model and the simplicity of the model.

**Table 3:** A: Risk Of ROP Associated with Each SNP In Infants with BDP (Unadjusted). B: Risk Of ROP Associated with Each SNP Adjusted for Gestational Age and Duration of Oxygen Use in Infants With BPD

A												
	Additive (Allele Dosage) Risk Model				Dominant Risk Model				Recessive Risk Model			
	Wt → μ	OR	95% CI	*p* value	Wt vs μ/*	OR	95% CI	*p* value	Wt/* vs μ/μ	OR	95% CI	*p* value
***SFTPA1***												
rs1059047	T → C	1.46	[0.41–5.21]	0.58	T/T vs C/*	1.86	[0.29–11.90]	0.48	T/* vs C/C	1.23	[0.15–9.97]	0.85
rs1136450	G → C	1.65	[0.67–4.04]	0.19	G/G vs C/*	1.97	[0.52–7.49]	0.24	G/* vs C/C	1.87	[0.39–9.12]	0.37
rs1136451	A → G	1.50	[0.43–5.25]	0.51	A/A vs G/*	1.50	[0.43–5.25]	0.51	A/* vs G/G		*no G/G*	
rs1059057	A → G	1.58	[0.24–10.61]	0.74	A/A vs G/*	1.58	[0.24–10.61]	0.74	A/* vs G/G		*no G/G*	
rs4253527	C → T	2.40	[0.51–11.26]	0.18	C/C vs T/*	2.40	[0.51–11.26]	0.18	C/* vs T/T		*no T/T*	
***SFTPA2***												
rs1059046	A → C	1.36	[0.56–3.30]	0.47	A/A vs C/*	1.54	[0.42–5.61]	0.49	A/* vs C/C	1.41	[0.27–7.26]	0.88
rs17886395	G → C	2.23	[0.74–6.78]	0.093	G/G vs C/*	3.06	[0.84–11.14]	0.052	G/* vs C/C	1.05	[0.06–18.05]	0.97
rs1965707	C → T	4.85	[1.40–16.77]	**0.0036**	C/C vs T/*	5.00	[1.35–18.56]	**0.0069**	C/* vs T/T	∞	*all T/T affected*	
rs1965708	C → A	2.47	[0.72–8.54]	0.089	C/C vs A/*	2.40	[0.64–9.03]	0.13	C/* vs A/A	∞	*all A/A affected*	
***SFTPD***												
rs721917	T → C	1.00	[0.39–2.54]	0.99	T/T vs C/*	1.41	[0.27–7.26]	0.88	T/* vs C/C	0.78	[0.20–3.11]	0.73
rs2243639	A → G	1.60	[0.67–3.82]	0.21	A/A vs G/*	2.64	[0.76–9.18]	0.077	A/* vs G/G	1.60	[0.67–3.82]	0.29

B								
	Additive Risk Model (Allele Dosage)				Dominant Risk Model			
	Wt → μ	OR	95% CI	*p* value	Wt/Wt vs */μ	OR	95% CI	*p* value
***SFTPA1***								
rs1059047	Additive T → C	1.79	[0.31–10.25]	0.48	T/T vs C/*	2.09	[0.21–21.08]	0.52
rs1136450	Additive G → C	1.75	[0.53–5.73]	0.27	G/G vs C/*	1.51	[0.24–9.56]	0.19
rs1136451	Additive A → G	1.79	[0.31–10.28]	0.48	A/A vs G/*	1.79	[0.31–10.28]	0.48
rs1059057	Additive A → G	3.90	[0.20 - 77.56]	0.28	A/A vs G/*	3.90	[0.20–77.56]	0.28
rs4253527	Additive C → T	2.56	[0.30 - 21.63]	0.31	C/C vs T/*	2.56	[0.30–21.63]	0.31
***SFTPA2***								
rs1059046	Additive A → C	2.32	[0.65–8.31]	0.12	A/A vs C/*	2.91	[0.49–17.26]	0.16
rs17886395	Additive G → C	3.33	[0.70–15.75]	0.070	G/G vs C/*	6.53	[0.87–49.24]	**0.027**
rs1965707	Additive C → T	9.10	[1.23–67.15]	**0.0070**	C/C vs T/*	9.80	[1.28–75.13]	**0.0085**
rs1965708	Additive C → A	2.88	[0.55–15.04]	0.13	C/C vs A/*	2.90	[0.53–15.93]	0.15
***SFTPD***								
rs721917	Additive T → C	0.47	[0.11–1.93]	0.20	T/T vs C/*	0.17	[0.01–2.43]	0.80
rs2243639	Additive G → A	1.72	[0.54–5.51]	0.29	G/G vs A/*	2.01	[0.39–10.46]	0.34

Each individual SNP was evaluated under each of three alternative inheritance patterns of risk: Additive (allele dosage), Dominant and Recessive. The wildtype (Wt) allele was determined as the most common allele in infants that did not have ROP. *OR* Odds Ratio, *CI* Confidence Interval, “p value” is from a Likelihood Ratio test whether adding the SNP to the risk model for ROP offers a significant improvement in goodness-of-fit. These are “unadjusted” results, i.e., neither gestational age nor days on O_2_ are included in the risk model. * indicates a wildcard representing any other possible tested nucleotide for the second allele; *p* value ≤ 0.05 are significant and highlighted in bold font; *GA* Gestational Age, *DO*_2_ days on oxygen, OR Odds Ratio, CI Confidence Interval.

Each individual SNP was evaluated under each of two alternative inheritance patterns of risk: Additive (Allele dosage) and Dominant. The wildtype (Wt) allele was determined as the most common allele in infants that did not have ROP. “p value” is from a Likelihood Ratio test whether adding the SNP to the risk model for ROP offers a significant improvement in goodness-of-fit. These are “adjusted” results, i.e., both GA and DO_2_ are included in the risk model; the Likelihood Ratio tests compare ROP = GA + DO_2_ vs ROP = SNP + GA + DO_2_. *p* value ≤ 0.05 are significant and highlighted in bold font; GA Gestational Age, DO_2_ days on oxygen, OR Odds Ratio, CI Confidence Interval.

**Table 4:** Akaike’s Information Criterion.

Risk model for rs1965707	AIC
Codominant (C/C, C/T, T/T)	56.44
Additive (allele dosage: C/C, [C/T = (C/C + T/T)/2], T/T)	**54.83**
Dominant (C/C, */T)	55.96
Recessive (C/*, T/T)	59.35

The best model has lowest AIC. *The solution for the recessive model was unstable so this AIC value is likely an approximate

Considering the Likelihood Ratio test results and the AIC results, the “Best” risk model for rs1965707 is the Additive (Allele dosage) model. The incidence of ROP in the heterozygote C/T was 67% and homozygote T/T was 100%, as expected under an allele dosage model; the incidence of ROP in the homozygote C/C was 32%.

The SNP rs17886395, noted as significantly associated with ROP but only under the Dominant model (*p* = 0.026) in the complete cohort, is not only marginally significant (*p* = 0.052) under this model, but is less significant (*p* = 0.093) under the Additive model.

Including GA and DO_2_ treatment in the analyses made little substantive change to these results in general (Table 3B). SFTPA2 rs1965707 continued to show significant association with risk for ROP under both Additive (*p* = 0.007) and Dominant (*p* = 0.0085) models. However, it was again noted that all the SFTPA2 SNPs shifted towards significance with the inclusion of these covariates. While testing for most SNPs remained marginal, rs17886395 now showed significant association with risk for ROP under the Dominant model (*p* = 0.027) and was now marginal (*p* = 0.070) under the Additive model.

The SFTPA1 SNP rs1059057 showed a significant association with ROP with the inclusion of the covariates under both Additive and Dominant models in the complete cohort; however, it was not significant in the BPD cohort.

### Association of haplotypes with ROP

SFTPA1 haplotypes were analyzed using additive and dominant risk models (Wt = 6A^2^), both with and without covariates as shown in Table 5, but none showed evidence of significant association of these haplotypes with risk of ROP. SFTPA2 haplotypes were similarly analyzed using additive and dominant risk models (Wt = 1 A^0^) without covariates, but none showed evidence of a significant association of these haplotypes with the risk of ROP. However, when analyzed including covariates GA and duration of oxygen treatment in the analyses, the Additive (*p* = 0.083) model remains marginally significant, but the Dominant (*p* = 0.11) is no longer even marginally significant.

**Table 5:** Risk of ROP associated with selected haplotypes adjusted for gestational age and duration of oxygen use in infants with BPD

Additive risk (allele dosage) model				Dominant risk model			
**SP-A1**							
**Model terms**	OR	95% CI	*p* value	**Model terms**	OR	95% CI	*p* value
6A^2^/6A^2^ → 6A^2^/6A* → 6A*/6A*	1.64	[0.32–8.44]	0.55	6A^2^/6A^2^vs */*	1.34	[0.20–9.04]	0.76
GA	0.52	[0.26–1.08]	0.080	GA	0.53	[0.26–1.09]	0.085
Days on O2	1.03	[0.99–1.06]	0.16	Days on O2	1.03	[0.99–1.06]	0.16
		LR =	0.55			LR =	0.72
**SP-A2**							
**Model terms**	OR	95% CI	p value	**Model terms**	OR	95% CI	p value
1A^0^/1A^0^ → 1A^0^/1A* → 1A*/1A*	2.91	[0.66–12.77]	0.16	1A^0^/1A^0^ vs */*	4.21	[0.50–35.34]	0.18
GA	0.39	[0.16–0.93]	**0.033**	GA	0.38	[0.16–0.92]	**0.031**
Days on O2	1.04	[1.00–1.08]	0.061	Days on O2	1.04	[1.00–1.08]	0.070
		LR =	0.083			LR =	0.11

Haplotypes were evaluated under each of two alternative inheritance patterns of risk: Additive (allele dosage) and Dominant. The base haplotype (6A^2^ and 1A^0^, respectively) were determined as the most common haplotype in infants that did not have ROP. “*p* value” is from a Wald’s test whether OR is significantly different from OR = 1 except were indicated by “LR =” where “*p* value” is from a Likelihood Ratio test whether adding the haplotype to the risk model for ROP offers a significant improvement in goodness-of-fit. These are “adjusted” results, i.e., both GA and DO_2_ are included in the risk model; the Likelihood Ratio tests compare ROP = GA + DO_2_ vs ROP = Haplotype + GA + DO_2_; Wald tests are from the latter model. These are “adjusted” results, i.e., both GA and DO_2_ are included in the risk model; the Likelihood Ratio tests compare ROP = GA + DO_2_ vs ROP = SNP + GA + DO_2_. * indicates a wildcard representing any other possible tested haplotype for the second allele; *p* value” ≤ 0.05 are significant and highlighted in bold font; GA Gestational Age; DO_2_ days on oxygen, OR Odds Ratio, CI Confidence Interval

### Gene × environment interaction

In Supplemental Table S5, an extended version of Table 3B, we noted a switch in significance, per the Wald tests, of the covariates from DO_2_ to GA, suggesting an interaction effect between SFTPA2: rs1965707 and the covariates. The was investigated further by first fitting the covariate-only model (ROP = GA + DO2) in each genotype sub-group Wt/Wt and */μ for each SNP (see Supplemental Table S6). In these results, we noted that for rs1965707 the OR for GA showed a marked change from 0.07 [0.01–0.85], *p* = 0.037 in the Wt/Wt sub-group to 0.77 [0.37–1.62], *p* = 0.49 in the */μ sub-group, further indicating an interaction effect. Sub-group differences in the GA effect also were seen for rs1059046 (OR = 0.36 vs OR = 0.62) and rs17886395 (OR = 0.95 vs OR = 0.12), although these did not reach significance within their respective sub-groups (by Wald tests). For each of these three SNPs (all in SFTPA2: rs1059046, rs17886395, rs1965707) a SNP by GA interaction effect was formally tested (Likelihood Ratio (LR) comparing the goodness-of-fit of the model with the interaction to the model without the interaction: [ROP = SNP + GA + DO2 vs ROP = SNP + GA + DO2 + SNP.GA]. A stable result was achieved only for [rs1965707.GA] where LR p value = 0.0079. To improve stability of the fitted model, “GA” was replaced with “preterm” (extreme preterm: GA < 28, very preterm: GA ≥ 28; see Table 1), i.e., the Likelihood Ratio was now [ROP = SNP + preterm + DO2 vs ROP = SNP + preterm + DO2 + SNP.preterm]. For the three SNPs, the LR results are:

1. rs1059046.preterm: LR p value = 0.62
2. rs17886395.preterm: LR p value = 0.061
3. rs1965707.preterm: LR p value = 0.0035

In summary, there is a significant interaction between SFTPA2: rs1965707 and GA, where GA is very protective against ROP in SFTPA2: rs1965707 C/C (the wildtype) but is relatively not protective in T/C or T/T.

## Discussion

**Fig. 3:**
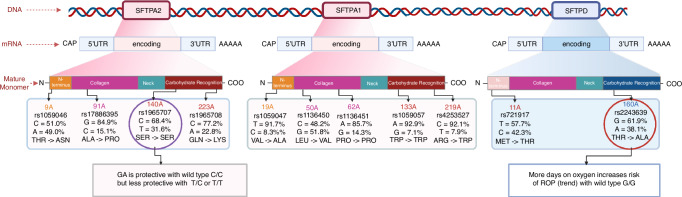
Surfactant protein nucleotide variants with their frequencies in study subjects, followed by resultant amino acids. Substitutions of significance are denoted with their association of risk for ROP. These variants may impact the function of the protein depending on the region of the protein monomer affected. This may include changes in folding, receptor binding and other protein-protein interactions. *UTR* untranslated region, *SFTPA* surfactant protein A, *SFTPD* surfactant protein D, *MBL* mannose binding lectin, *mRNA* messenger RNA, *GA* gestational age, *ROP* retinopathy of prematurity, *THR* threonine, *ASN* asparagine, *ALA* alanine, PRO proline, *SER* serine, *GLN* glutamine, *LYS* lysine, *VAL* valine, *LEU* leucine, *TRP* tryptophan, *MET* methionine. Created in BioRender. Bhatti, F. (2025) https://BioRender.com/tblrmjc.

In this study, we report the novel association of polymorphisms in the human surfactant protein genes SFTPA1, SFTPA2, and their associated haplotypes, with the odds of developing ROP while controlling for GA, oxygen exposure and lung disease in preterm infants. We did not find significant associations with SNPs related to SFTPD. SFTPA1 SNPs did not alter the odds of developing ROP when controlling for GA and duration of oxygen treatment. However, when considering *SFTPA2* SNP, rs1965707, increasing GA is very protective against ROP in individuals carrying the C/C genotype (the wildtype), but is relatively not protective in those with T/C or T/T. Furthermore, increasing GA is protective against ROP in the presence of the SFTPA2 wildtype haplotype variant 1A^0^. A summarization of the SNP associations along with their relative locations on the genes and amino acid substitutions is shown in Fig. 3. While the SP-A1/ SP-A2 haplotype 6A^2^/1A^0^ and the SP-D/SP-A2 locus DA160(A)/SP-A2(1A^1^) confer protection in lung disease, we did not find a protective effect of 6A^2^ in this study. Complete haplotype analysis was not possible secondary to small numbers; however, our observed SNP data indicate a plausible interplay between the prevalent SFTPA2 haplotype, encompassing rs1965707, and GA in modulating the susceptibility to ROP, potentially through mechanisms affecting retinal vascular development and response to oxidative stress.

Surfactant proteins A and D have a sophisticated structure that is highly dependent on the assembly of individual protein monomers. In primates, SP-A is encoded by two genes resulting from a duplication, SFTPA1 and SFTPA2, which, along with SFTPD, are located on chromosome 10. The translated SP-A protein monomer has four distinct regions, as shown in Fig. 1, with differing roles in SP-A target effects.^7^ The mature surfactant protein A complex requires two SP-A1 gene products and one SP-A2 product per trimer.^38^ The final conformation may therefore differ because of SNP variants resulting in variable affinity for their receptors and downstream immunomodulatory pathways. The SNPs all correspond to specific amino acid locations. SP-A1 and SP-A2 have several reported polymorphisms with associated haplotypes.^24^ SP-D also has two predominant polymorphisms: rs721917 (T → C, Met11Thr) and rs2243639 (A → G and Ala160Thr), each having a frequency exceeding 20%.^25^ These variants are shown above in Fig. 2. It is important to note that not every known and/or reported SP-A and SP-D variant was included in our analysis. The SNPs and variants analyzed here were chosen for their frequency and reported associations with diseases seen in preterm infants.

### SFTPA1

Our data show that in infants with and without ROP, there was no association of the odds of developing ROP with single-variant analysis with the SFTPA1 SNPs considered here, nor were any of the 6A^2^ allelic variants significantly associated with the odds of developing ROP. This suggests that while surfactant protein A is crucial for pulmonary function, genetic variations within SFTPA1 may not exert a primary influence on ROP development. This finding underscores the complexity of ROP pathogenesis and suggests that other genetic or environmental factors may play more prominent roles in modulating disease risk.

### SFTPA2

Our study identified rs1965707 in SFTPA2 as significantly associated with ROP risk. Our results show that the C/C genotype of rs1965707 is associated with a 32% incidence of ROP, while heterozygotes (C/T) have a 67% incidence, and homozygotes (T/T) have a 100% incidence. This dosage-dependent effect suggests a role for rs1965707 in ROP susceptibility. This SNP is intronic in SFTPA2, and it is likely that this SNP has a regulatory role in alternative splicing. This may have functional implications, such as in mRNA splicing, stability, and translation, as well as potential influence on the protein’s structure, secretion, and interaction with immune cells within the retinal microenvironment. The identified association with rs1965707 was further substantiated by interaction analyses, which indicated a significant interplay between this SNP and gestational age in modulating ROP risk. Gestational age is highly protective against ROP in infants with the C/C genotype, but not in those with C/T or T/T genotypes.

The rs1965707 SNP is located at amino acid 140 and mapped to the carbohydrate recognition domain of this C-type lectin. Its function is to bind carbohydrates to microorganisms and initiate innate defense mechanisms.^39^ The CRD is critical for the binding of SP-A to a host of receptors, including key macrophage receptors.^7,40–46^ These include cell surface or endoplasmic reticulum membrane-bound C1q (calreticulin), CD14, Signal-Inhibitory Regulatory Protein alpha (SIRPα), CR3 or CD11b, and SPR-210. The binding of SP-A to these receptors can variably activate macrophage activity/function or can upregulate the expression of cytokines and interleukins. Functional macrophages are critical in physiological blood vessel development and have been shown to modulate retinal angiogenesis. We therefore surmise that the SNP rs1965707 impacts normal endothelial cell function and angiogenesis and may play a protective role against retinal vascular disease.

A critical mechanistic consideration is the localization of this SNP within the carbohydrate recognition domain, which changes a cytosine to a thymine, but does not change the amino acid from serine in the translated protein. While it is unclear why this SNP is associated with a greater risk of developing ROP, as the mechanisms have not been yet studied, it is possible that this change results in differential regulation of mRNA expression or protein translation mechanisms.

Lower GA was protective against ROP with rs17886395 in the dominant risk model when adjusting for GA and DO_2_. However, the subsequent interaction effects did not show any statistically significant effects.

This SNP is also located at amino acid 91 within the collagen-like domain. The presence of cytosine instead of guanidine at this location is associated with increased odds of developing ROP. This cytosine changes the amino acid from an alanine to a proline. Because this part of the structure is dominated by α-helix, the presence of a proline may disrupt the folding of SP-A2 and reduce its ability to associate with other surfactant proteins.

The representation of the *SFTPA2* SNP rs1059046 *was a key mechanistic clue in our results*. The non-wild-type variant was represented by threonine instead of asparagine at aa9. This SNP is in the N-terminus (Fig. 3), a non-collagenous, cysteine-rich region critical for oligomerization, which occurs by disulfide bridging.^47^ The inter-chain linkages are formed by cysteine residues.^48^ Cys85 has been shown to mediate macrophage activity; however, this may be due to a more general cellular cytoskeleton-driven mechanism.^49,50^ Our unpublished data in rodents and human retinal endothelial cells suggest that actin expression and organization within endothelial cells are modulated by SP-A. It is thus conceivable that variants involving cysteine residues may be critical for the cytoskeleton and cell mobility and movement. The C-type lectin domain associates with the collagen body through a strong hydrophobic interaction via the a-helical bundle forming the neck region. Within it, Gly-X-Y repeats bind in a triple helix, similar to a zipper-like structure. The carbohydrate-binding or lectin domain is what defines the function of all collectin proteins by virtue of binding to a variety of carbohydrates and lipids. It is important in mediating immune response, since it recognizes carbohydrate epitope moieties of different sizes and shapes from multiple microorganisms when polymerized in their tridimensional structure.^51^ This will drive how the final assembled product behaves in a disease-specific context. For example, it has been reported that the cysteine residue at aa85 plays an important role in driving the phagocytic activity of alveolar macrophages^52^ and that the SP-A variant with Arg85 enhanced bacterial phagocytosis compared to Cys85.

### SFTPA variants

An allelic variant is the DNA sequence at a specific chromosomal location, which presents as a variant, or SNPs, in a gene. Any given gene can have multiple different alleles. A haplotype is a set of alleles on a single chromatid that are physically bound and may be statistically associated with one another.

**Fig. 4:**
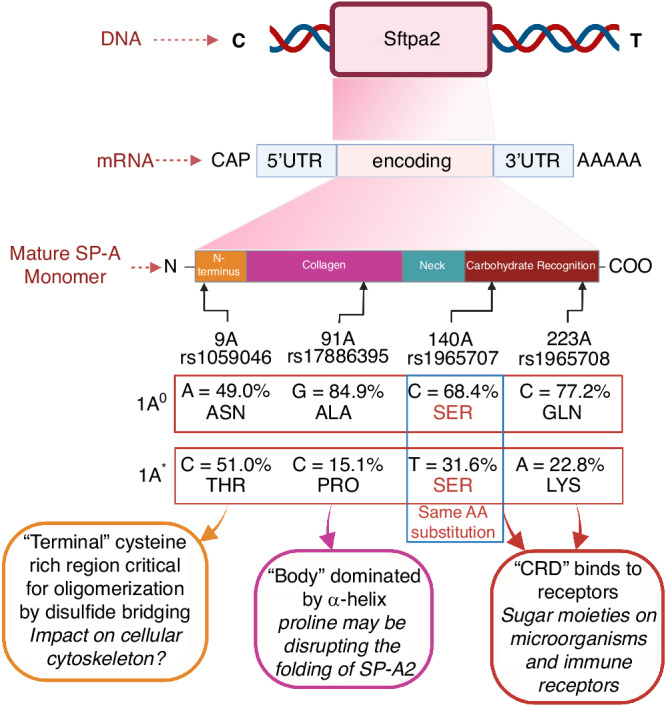
SFTPA2 allelic variants with frequency of nucleotide substitutions representing the 1A^0^ variant of SFTPA2 in both the wild-type and non-wild-type variants. When looking at the most frequent SNPs represented in the alternative 1A^*^ variant (non-wild type) it is marked by threonine in the n-terminus, proline in the collagen-like region and lysine in the CRD. Predicted protein confirmational changes are denoted for each variant of significance. UTR untranslated region, mRNA messenger RNA, SFTPA surfactant protein A, CRD carbohydrate recognition domain, ASN asparagine, THR threonine, ALA alanine, PRO proline, SER serine, GLN glutamine, LYS lysine. Created in BioRender. Bhatti, F. (2025) https://BioRender.com/tblrmjc.

For SFTPA2 haplotypic variants, the wild-type variant in our population is 1A^0^. In analyzing the association of this variant with risk of ROP, when including the covariates GA and DO_2_ in the analyses, the additive (*p* = 0.083) model showed marginal significance in altering odds of ROP. The wild type and non-wild type substitutions of the nucleotides and amino acids occur in the specified locations: aa9-aspargine, aa91-alanine, aa140-serine and aa223-glutamine as shown in Fig. 4.

Prior studies in preclinical models have shown that differences among SP-A1 and SP-A2 alleles drive their ability to stimulate TNF-alpha production in THP-1 cells, with greater TNF-alpha production from SP-A2 vs SP-A1 alleles^53^ or exert different effects on the macrophage proteome in a sex-dependent manner.^54^ This included differences in the expression of actin and macrophage motility. Our group has found similar SP-A-mediated differences in actin expression in endothelial cells. This leads us to believe that SP-A may directly or indirectly drive endothelial cell function and vascular growth. We have previously shown that SP-A is expressed in the mouse retina^23^ and is associated with a pro-angiogenic phenotype of the retinal vasculature.^22^ Thus, not only does the expression of total SP-A or SP-D protein have the potential to mediate immune responses, but there may be an equally important effect of the SNP-variant-mediated downstream signaling pathways.

### SFTPD

With SFTPD, we did not observe any significant correlations between the SNPs and odds of ROP in all infants, or in infants with BPD (with and without adjustment). Supplemental Table 4 shows the modeling with Wald’s testing for infants with BPD to compare models for best fit. An interesting observation is that rs2243639 appeared to have an interactive effect with DO_2_, in that greater DO_2_ is associated with ROP in the presence of this SNP in dominant risk modeling; however, the LR is not significant, indicating a lack of goodness of fit. It is possible that the small sample size made it difficult to achieve statistical significance. However, when reviewing the location of this SNP, it is within the CRD of the mature SP-D protein. The dominant risk model is represented by G/G, which is the wildtype. It has been shown that adults with A/G and A/A genotypes are at increased risk of chronic obstructive pulmonary disease (COPD).^55^ A study looking at RSV in infants^8^ showed a significant association with rs721917, but not with rs2243639. This suggests that overall, SP-D may play a greater role in varying severity of lung disease and oxygen duration and may not have an association with vascular pathways per se, although studies with larger sample sizes are needed to say this conclusively.

While we report several novel and key findings related to surfactant protein biology, we also note several limitations to our study. Statistical, not Functional: While our study has identified a significant association between the SFTPA2 rs1965707 polymorphism and ROP, dissecting the precise molecular mechanisms that mediate this relationship necessitates further investigation. Small Sample Size: The relatively modest sample size may have limited the statistical power to detect associations with smaller effect sizes. The absence of significant associations with SFTPA1 SNPs, especially rs1136450, may be reversed with a larger sample size. This also limited exploration of the heterogeneity of ROP phenotypes. Limited Generalization: We found no impact of ethnic background in analyzing the association of SNP variants with the risk of developing ROP. However, the study population was from one NICU only, which may limit the generalizability of the findings to other populations; future studies with larger, more diverse cohorts are warranted to validate these findings and to identify additional genetic variants that may contribute to ROP susceptibility across different ethnic backgrounds. Limited Covariates: While we adjusted for several known risk factors for ROP, residual confounding by unmeasured or poorly measured variables cannot be entirely ruled out; future studies incorporating comprehensive data on perinatal exposures, clinical management strategies, and longitudinal ophthalmologic outcomes are needed to refine our understanding of the complex interplay of factors contributing to ROP pathogenesis and to improve risk prediction models for this condition. Single-timepoint analysis was performed of the variants from blood, and plasma surfactant protein concentrations could not be determined. This obscures possible epigenetic contributions to the final disease phenotype. Limited Candidate Genes: This study focused on a limited number of candidate genes based on their known roles in lung development and oxygen homeostasis; future genome-wide association studies may identify novel genetic loci that contribute to ROP susceptibility beyond the SFTPA2 gene.

However, our study opens the question of SNP location-specific effects on the final protein confirmation and activity in terms of how the surfactant proteins interact with endothelial and other cell types in the retina. Most profoundly, it determines that surfactant gene SNP products differ in their disease effect in the presence or absence of oxygen, which is highly suggestive of a direct impact of surfactant proteins A and D on endothelial cell function, which is independent of their impact on pulmonary inflammatory disease and macrophage function.

## Conclusions

By studying SNPs and haplotypes of surfactant protein genes in a cohort of preterm infants with BPD and ROP, we identified important and novel associations of SFTPA1/SFTPA2 polymorphisms impacting the odds of developing ROP. The identified SNPs encode amino acid substitutions impacting protein folding, oligomerization, macrophage activation, and potentially expression of mature proteins. Gestational age was protective against ROP in the presence of the SFTPA2 wildtype allelic variant 1A^0^. Taken together, these findings suggest a direct effect of SP-A on vascular morphology, including endothelial cell function, angiogenesis, and response of vascular growth factors to inflammatory signals. We conclude that these gene polymorphisms may regulate both expression levels and protein structure, thus influencing the effects of surfactant proteins on the retinal and pulmonary vasculature and development of ROP.

### Confirmation of the author’s publication and licensing rights

Science Suite Inc. dba BioRender (“BioRender”) has granted Faizah Bhatti permission to use this Completed Graphic in accordance with BioRender’s Terms of Service and Academic License Terms (“License Terms”). The corresponding author (FNB) holds open access publishing rights to this manuscript from the Elsevier Licence, of which she was senior author: CC BY-NC-ND 4.0 Attribution-Noncommercial-Noderivatives 4.0 International. Fig. 1 in this submitted manuscript is adapted from the publication: Vieira, F., Kung, J. W. & Bhatti, F. Structure, Genetics and Function of the Pulmonary Associated Surfactant Proteins a and D: The Extra-Pulmonary Role of These C Type Lectins. *Ann Anat* **211**, 184-201 (2017). Canonical URL https://creativecommons.org/licenses/by-nc-nd/4.0/.

## Supplementary information

Supplementary Information

## Data Availability

The genomic reference datasets analyzed during the current study are publicly available in the dbSNP Database^56^ https://www.ncbi.nlm.nih.gov/snp/.
