# Supplementary material for: Surfactant proteins A and D nucleotide variants: association with retinal vascular disease
Source: Pediatr Res. 2025 Oct 16;99(5):2055–65. doi: 10.1038/s41390-025-04435-w (PMC12600029; doi:10.1038/s41390-025-04435-w)
Supplement: Supplementary file 1 — Supplementary Information [file 41390_2025_4435_MOESM1_ESM.pdf]

SUPPLEMENTARY DATA FILE 1

METHODS

Genotyping of Variants: After sample collection, DNA was extracted and the samples analyzed at the University of Indiana for polymerase chain reaction (PCR) based genotyping of the variants of interest. Several validated methods were used for determining the SNPs; these are outlined below:

Method 1 utilized TaqMan allelic discrimination assays with purified DNA. DNA samples were first diluted to 25 ng/µl and underwent qPCR with TaqMan Genotyping Master Mix (Cat. 4371355) and TaqMan assays specific to each SNP (see below). As per the protocol, 5 µl of master mix was used, 0.5 µl of 20X TaqMan Genotyping Assay was used, and 4.5 µl of diluted DNA was used for 10 µl reactions on a 384-well reaction plate (Cat. 4309849). Final mass of DNA in each 10 µl reaction was 112.5 ng. qPCR was run on QuantStudio 5 using Design and Analysis Software v1.5.2 and results were analyzed and called by hand. The program consisted of pre-PCR read (60 C for 30 seconds), PCR reads (95 C for 10 minutes, [95 C for 15 seconds and 60 C for 1 minute] x 40 times), and a post-PCR read (60 C for 30 seconds).

Method 2 utilized gene specific nested PCR in combination with TaqMan allelic discrimination assays. DNA samples were first diluted to 25 ng/µl and then underwent gene specific PCR. For SNPs on gene SP-A1, primers 68A and 326 were used; for SP-A2, primers 68A and 327 were used. PCR was done with 10µl of Platinum II Hot Start PCR Master Mix (Cat. 14001013), 0.4 µl of 10 µM forward primer, 0.4 µl of 10 µM of reverse primer, and 9.2 µl of diluted DNA for a 20 µl reaction. The reaction conditions were 94 C for 2 minutes, [94 C for 15 seconds, 58 C for 15 seconds, and 68 C for 1 minute] thirty-five times, and hold at 4 C. The product of this reaction was diluted 1:100 and then underwent qPCR using TaqMan Genotyping Master Mix (Cat. 4371355) and TaqMan assays specific to each SNP (see below). 5 µl of master mix was used, 0.5 µl of 20X TaqMan Genotyping Assay was used, and 4.5 µl of product was used for 10 µl reactions on a 384-well reaction plate (Cat. 4309849). qPCR was run on

QuantStudio 5 using Design and Analysis Software v1.5.2 and results were analyzed and called by hand. The program consisted of pre-PCR read (60 C for 30 seconds), PCR reads (95 C for 10 minutes, [95 C for 15 seconds and 60 C for 1 minute] x 40 times), and a post-PCR read (60 C for 30 seconds).

Method 3 utilized restriction fragment length polymorphism (RFLP) analysis after gene-specific and amino acid specific PCR (modified from DiAngelo et. al 1999). DNA samples were first diluted to 25 ng/μl and then underwent gene specific PCR, as specified above. The product of this reaction was diluted 1:100 and then underwent a second PCR to amplify the region around the SNP. This was done by using 10μl of Platinum II Hot Start PCR Master Mix (Cat. 14001013), 0.4 μl of 10 μM forward primer, 0.4 μl of 10 μM of reverse primer, 1 μl of diluted DNA, and 8.2 μl of water for a 20 μl reaction. The reaction conditions were 94 C for 2 minutes, [94 C for 15 seconds then 58 C for 15 seconds] 35 times and hold at 4 C. 5 μl of the product then underwent a restriction reaction using 0.5 μl of the enzyme, 2 μl of the 10X NEBuffer, 3.3 μl of loading dye, and 9.2 μl of water for a 20 μl reaction. This reaction was carried out at 37 C for 15 minutes then the enzyme was deactivated at 65 C for 20 minutes. The restriction enzyme cut at the SNP site if a certain nucleotide was present. The product of this restriction reaction was then run on a 2% agarose gel in 0.5X TBE buffer using 1X of SYBR Safe (Cat. S33102). A 50 bp ladder was run with each row of samples to check the length of the bands. Gels were imaged using a BioRad ChemiDoc using ImageLab.

Method 4 utilized sequencing after gene-specific and amino acid specific PCR. DNA samples were first diluted to 25 ng/μl and then underwent gene specific PCR, as specified above. For SNPs on gene SP-A1, primers 68A and 326 were used; for SP-A2, primers 68A and 327 were used. The product of this reaction was diluted 1:100 and then underwent a second PCR to amplify the region around the SNP as specified above. The product was then sent to Eurofins with the respective forward primers to be sequenced. The results were then analyzed and called by hand.

Gene	SNP ID	aa Position	SNP (Probe)	Method	Qpcr Probe Cat. #	Primers Used (In Order) And Restriction Enzymes
**SP-A1**	*rs1059047*	19	T/C	1	C_7433969_10	N/A
	rs1136450	50	C/G	3	N/A	68A & 326; 787 & 765; DdeI
	rs1136451	62	A/G	2	C_175676233_20	68A & 326
	rs1059057	133	A/G	1	ANGZYDA	N/A
	rs4253527	219	C/T	2	ANH6TW7	68A & 326
**SP-A2**	*rs1059046*	9	C/A	4	N/A	68A & 327; SPA2aa9For & SPA2aa9Rev*
	rs17886395	91	G/C	4	N/A	68A & 327; SPA2aa91For & SPA2aa91Rev*
	rs1965707	140	C/T	2	ANRWXUH	68A & 327
	rs1965708	223	C/A	2	C_26677208_10	68A & 327
**SP-D**	*rs721917*	11	T/C	1	C_26726205_30	N/A
	rs2243639	160	A/G	1	C_1362980_10	N/A

SM Table 1. Summary of Methods. SNP IDs with the resultant amino acid variants with their position. Method refers too PCR and identification approach as outlined in the text. PCR probes and enzymes are also reported. SNPs listed in the SNP (probe) column are defined as the "reference" for this study.

*SPA2aa9For: 5'CACAGTGGGGGAGATGTTGG3'

*SPA2aa9Rev: 5'GCCAGGGTCTCCTTTGACAC3'

*SPA2aa91For: 5'GCATCGAGTCTCACTAGCTC3'

*SPA2aa91Rev: 5'GTTGAGCCAAATGCCCTTGG3

SUPPLEMENTAL DATA TABLES SD2

Gene	SNP	Genotype	Infants without ROP (n=36)	Infants with ROP (n=23)	% ROP within genotype
**SFTPA1**					
	rs1136450	C/C	7 (19.4)	5 (21.7)	41.7%
		C/G	17 (47.2)	13 (56.5)	43.3%
		G/G	12 (33.3)	5 (21.7)	29.4%
	rs1136451	A/A	25 (69.4)	13 (56.5)	34.2%
		G/A	9 (25.0)	10 (43.5)	52.6%
		G/G	2 (5.6)	0	0%
	rs1059047	C/C	3 (9.4)	3 (13.0)	50.0%
		T/C	20 (55.6)	12 (52.2)	37.5%
		T/T	8 (22.2)	2 (8.7)	20.0%
	rs1059057	A/A	33 (91.7)	19 (82.6)	36.5%
		A/G	3 (8.3)	4 (17.4)	57.1%
		G/G	0	0	-
	rs423527	C/C	30 (83.3)	17 (73.9)	36.2%
		C/T	5 (13.9)	6 (26.1)	54.5%
		T/T	1 (2.8)	0	0%
**SFTPA2**					
	rs1059046	A/A	14 (38.9)	6 (26.1)	30.0%
		A/C	15 (41.7)	13 (56.5)	46.4%
		C/C	7 (19.4)	4 (17.4)	36.4%
	rs17886395	C/C	2 (5.6)	1 (4.5)	33.3%
		C/G	7 (19.4)	11 (50.0)	61.1%
		G/G	27 (75.0)	10 (45.5)	27.0%
	rs1965707	C/C	25 (69.4)	8 (34.8)	24.2%
		C/T	9 (25.0)	13 (56.5)	59.1%
		T/T	2 (5.6)	2 (8.7)	50.0%
	rs1965708	A/A	1 (2.8)	1 (4.3)	50.0%
		C/A	9 (25.0)	9 (39.1)	50.0%
		C/C	26 (72.2)	13 (56.5)	33.3%
**SFTPD**					
	rs721917	C/C	8 (22.2)	6 (26.1)	42.9%
		T/C	21 (58.3)	14 (60.9)	40.0%
		T/T	7 (19.4)	3 (13.0)	30.0%
	rs2243639	A/A	4 (11.4)	3 (13.0)	42.9%
		A/G	13 (37.1)	12 (52.2)	48.0%
		G/G	18 (51.4)	8 (34.8)	30.8%

Supplemental Table 1: Genotype frequencies for individual SNPs in infants with and without ROP, as well as incidence of ROP in infants with the genotype. Counts for all infants, with and without BPD.

Gene	Genotype	Infants without ROP (n=36)	Infants with ROP (n=23)	% ROP within Genotype
**SFTPA1**				
	6A/6A2	1 (2.8%)	2 (8.7%)	66.7%
	6A/6A3	4 (11.1%)	3 (13.0%)	42.9%
	6A/6A4	1 (2.8%)	0	0%
	6A2/6A2	13 (36.1%)	5 (21.7%)	27.8%
	6A2/6A3	11 (30.6%)	6 (26.1%)	35.3%
	6A2/6A4	2 (5.6%)	2 (8.7%)	50.0%
	6A3/6A3	1 (2.8%)	2 (8.7%)	66.7%
	6A3/6A4	2 (5.6%)	3 (13.0%)	60.0%
	6A4/6A4	1 (2.8%)	0	0%
	6A	6 (8.3%)	5 (10.9%)	
	**6A2**	**40 (55.6%)**	**20 (43.5%)**	
	6A3	19 (26.4%)	16 (34.8%)	
	6A4	7 (7.5%)	5 (10.9%)	
**SFTPA2**				
	1A/1A	1 (2.8%)	0	0%
	1A/1A0	3 (8.3%)	4 (17.4%)	57.1%
	1A/1A1	0	1 (4.3%)	100.0%
	1A/1A2	1 (2.8%)	0	0%
	1A/1A3	1 (2.8%)	1 (4.3%)	50.0%
	1A/1A5	1 (2.8%)	1 (4.3%)	50.0%
	1A0/1A0	13 (36.1%)	4 (17.4%)	23.5%
	1A0/1A1	5 (13.9%)	4 (17.4%)	44.4%
	1A0/1A2	5 (13.9%)	0	0%
	1A0/1A3	1 (2.8%)	2 (8.7%)	66.7%
	1A0/1A5	1 (2.8%)	4 (17.4%)	80.0%
	1A1/1A1	1 (2.8%)	1 (4.3%)	50.0%
	1A1/1A2	1 (2.8%)	0	0%
	1A1/1A5	1 (2.8%)	1 (4.3%)	50.0%
	1A2/1A2	1 (2.8%)	0	0%
	1A	8 (11.1%)	7 (15.2%)	
	**1A0**	**41 (56.9%)**	**22 (47.8%)**	
	1A1	9 (12.5%)	8 (17.4%)	
	1A2	9 (12.5%)	0	
	1A3	2 (2.8%)	3 (6.5%)	
	1A5	3 (4.2%)	6 (13.0%)	

Supplemental Table 2: Genotype and haplotype frequencies for SFTPA1 and SFTPA2 haplotypes in infants with and without ROP, as well as incidence of ROP in infants with the genotype. Most Frequent Allele (MFA) is indicated in bold. Counts for all infants, with and without BPD.

	Additive Risk Model (Allele Dosage)				Dominant Risk Model				
	**Wt → μ**	**OR**	**95% CI**	**p value**		**Wt vs μ/***	**OR**	**95% CI**	**p value**
SFTPA1									
rs1059047	T → C	2.01	[0.67 - 6.02]	0.21	rs1059047	T/T vs C/*	2.61	[0.49 - 14.00]	0.26
rs1136450	G → C	1.33	[0.63 - 2.85]	0.46	rs1136450	G/G vs C/*	1.80	[0.54 - 6.03]	0.34
rs1136451	A → G	1.27	[0.50 - 3.24]	0.62	rs1136451	A/A vs G/*	1.75	[0.59 – 5.19]	0.31
rs1059057	A → G	2.32	[0.47 - 11.47]	0.30	rs1059057	A/A vs G/*	2.32	[0.47 - 11.47]	0.30
rs4253527	C → T	1.37	[0.44 - 4.27]	0.59	rs4253527	C/C vs T/*	1.76	[0.49 - 6.34]	0.38
SFTPA2									
rs1059046	A → C	1.24	[0.59 - 2.60]	0.57	rs1059046	A/A vs C/*	1.80	[0.57 - 5.68]	0.31
rs17886395	G → C	2.28	[0.90 - 5.75]	0.082	rs17886395	G/G vs C/*	3.60	[1.16 - 11.13]	**0.026**
rs1965707	C → T	2.74	[1.11 - 6.76]	**0.028**	rs1965707	C/C vs T/*	4.26	[1.40 - 12.97]	**0.011**
rs1965708	C → A	1.76	[0.68 - 4.57]	0.24	rs1965708	C/C vs A/*	2.00	[0.67 - 6.01]	0.22
SFTPD									
rs721917	T → C	1.29	[0.56 - 2.97]	0.55	rs721917	T/T vs C/*	1.61	[0.37 - 6.98]	0.53
rs2243639	G → A	1.48	[0.68 - 3.24]	0.32	rs2243639	G/G vs A/*	1.99	[0.67 - 5.87]	0.22

Table S3: Risk of ROP Associated with Each SNP In All Infants (Unadjusted). Each individual SNP was evaluated under each of two alternative inheritance patterns of risk: Additive (allele dosage) and Dominant. The wildtype (Wt) allele was determined as the most common allele in infants that did not have ROP. “p value” is from a Likelihood Ratio test whether adding the SNP to the risk model for ROP offers a significant improvement in goodness-of-fit. “p value” ≤ 0.05 are considered to be significant and highlighted in bold font. These are “unadjusted” results, i.e., neither gestational age nor duration of oxygen treatment (days on O_2) are included as terms in the risk model. OR: Odds Ratio, CI: Confidence Interval.

	Additive Risk Model (Allele Dosage)					Dominant Risk Model			
	Model terms	OR	95% CI	p value		Model terms	OR	95% CI	p value
SFTPA1									
rs1059047 (LR=0.12)	Additive T → C	2.52	[0.62 - 10.25]	0.20	rs1059047 (LR=0.36)	T/T vs C/*	2.14	[0.31 - 14.73]	0.44
	GA	0.78	[0.49 - 1.25]	0.30		GA	0.78	[0.50 - 1.22]	0.28
	Days on O2	1.03	[1.00 - 1.05]	0.059		Days on O2	1.02	[1.00 - 1.05]	0.073
rs1136450 (LR=0.24)	Additive G → C	1.64	[0.61 - 4.42]	0.33	rs1136450 (LR=0.28)	G/G vs C/*	2.05	[0.44 - 9.57]	0.36
	GA	0.74	[0.47 - 1.16]	0.19		GA	0.74	[0.47 - 1.16]	0.19
	Days on O2	1.03	[1.00 - 1.05]	**0.025**		Days on O2	1.03	[1.00 - 1.05]	**0.029**
rs1136451 {LR=0.46)	Additive A → G	1.58	[0.43 - 5.80]	0.49	rs1136451 (LR=0.36)	A/A vs G/*	1.78	[0.43 - 7.31]	0.42
	GA	0.77	[0.50 - 1.19]	0.23		GA	0.77	[0.49 - 1.19]	0.23
	Days on O2	1.03	[1.01 - 1.05]	**0.013**		Days on O2	1.03	[1.01 - 1.05]	**0.015**
rs1059057 (LR=0.047)	Additive A → G	7.09	[0.76 - 66.09]	0.085	rs1059057 (LR=0.047)	A/A vs G/*	7.09	[0.76 - 66.09]	0.085
	GA	0.82	[0.52 - 1.29]	0.39		GA	0.82	[0.52 - 1.29]	0.39
	Days on O2	1.04	[1.01 - 1.06]	**0.0065**		Days on O2	1.04	[1.01 - 1.06]	**0.0065**
rs4253527 (LR=0.54)	Additive C → T	1.23	[0.24 - 6.24]	0.80	rs4253527 (LR=0.54)	C/C vs T/*	1.23	[0.24 - 6.24]	0.80
	GA	0.78	[0.50 - 1.20]	0.26		GA	0.78	[0.50 - 1.20]	0.26
	Days on O2	1.03	[1.00 - 1.05]	0.018		Days on O2	1.03	[1.00 - 1.05]	0.018
SFTPA2									
rs1059046 (LR=0.13)	Additive A → C	1.97	[0.69 - 5.60]	0.20	rs1059046 (LR=0.082)	A/A vs C/*	3.14	[0.69 - 14.37]	0.14
	GA	0.71	[0.44 - 1.13]	0.15		GA	0.69	[0.43 - 1.11]	0.13
	Days on O2	1.03	[1.00 - 1.05]	**0.026**		Days on O2	1.03	[1.00 - 1.05]	**0.026**
rs17886395 (LR=0.088)	Additive G → C	2.46	[0.74 - 8.26]	0.14	rs17886395 (LR=0.029)	G/G vs C/*	4.24	[0.95 - 19.01]	0.059
	GA	0.88	[0.52 - 1.48]	0.63		GA	0.90	[0.53 - 1.53]	0.69
	Days on O2	1.04	[1.01 - 1.07]	**0.013**		Days on O2	1.04	[1.01 - 1.07]	**0.012**
rs1965707 (LR=0.012)	Additive C → T	4.54	[1.15 - 17.91]	**0.031**	rs1965707 (LR=0.010)	C/C vs T/*	6.07	[1.26 - 29.15]	**0.024**
	GA	0.64	[0.40 - 1.03]	0.065		GA	0.64	[0.40 - 1.03]	0.064
	Days on O2	1.02	[1.00 - 1.05]	0.089		Days on O2	1.02	[1.00 - 1.04]	0.12
rs1965708 (LR=0.076)	Additive C → A	2.78	[0.74 - 10.46]	0.13	rs1965708 (LR=0.075)	C/C vs A/*	3.12	[0.72 - 13.51]	0.13
	GA	0.75	[0.49 - 1.13]	0.17		GA	0.75	[0.49 - 1.13]	0.17
	Days on O2	1.03	[1.00 - 1.05]	**0.017**		Days on O2	1.03	[1.00 - 1.05]	**0.018**
SFTPD									
rs721917 (LR=0.25)	Additive T → C	0.93	[0.32 - 2.76]	0.90	rs721917 (LR=0.55)	T/T vs C/*	0.56	[0.09 - 3.63]	0.55
	GA	0.78	[0.51 - 1.21]	0.27		GA	0.77	[0.50 - 1.18]	0.22
	Days on O2	1.03	[1.01 - 1.05]	**0.012**		Days on O2	1.03	[1.01 - 1.15]	**0.0099**
rs2243639 (LR=0.13)	Additive G → A	1.95	[0.72 - 5.30]	0.19	rs2243639 (LR=0.081)	G/G vs A/*	3.00	[0.71 - 12.74]	0.14
	GA	0.87	[0.57 - 1.34]	0.53		GA	0.87	[0.57 - 1.34]	0.54
	Days on O2	1.04	[1.01 - 1.06]	**0.0062**		Days on O2	1.04	[1.01 - 1.06]	**0.0054**

Table S4: Risk of ROP Associated with Each SNP Adjusted for Gestational Age and Duration of Oxygen Use in All Infants. Each individual SNP was evaluated under each of two alternative inheritance patterns of risk: Additive (allele dosage) and Dominant. The wildtype (Wt) allele was determined as the most common allele in infants that did not have ROP. “p value” is from a Wald’s test whether OR is significantly different from OR = 1, the p value indicated by “LR =” is from a Likelihood Ratio test whether adding the haplotype to the risk model for ROP offers a significant improvement in goodness-of-fit. “p value” ≤ 0.05 are considered to be significant and highlighted in bold font. These are “adjusted” results, i.e., both GA) and DO_2 are included in the risk model; the Likelihood Ratio tests compare ROP = GA + DO_2 vs ROP = SNP + GA + DO_2; Wald tests are from the latter model. GA: gestational age; DO2: days on O_2.

	Additive Risk Model (Allele Dosage)				Dominant Risk Model				
	Model terms	OR	95% CI	p value		Model terms	OR	95% CI	p value
SFTPA1									
rs1059047	Additive T → C	1.79	[0.31 - 10.25]	0.51	rs1059047	T/T vs C/*	2.09	[0.21 - 21.08]	0.53
(LR=0.48)	GA	0.58	[0.30 - 1.11]	0.100	(LR=0.52)	GA	0.56	[0.29 - 1.10]	0.091
	Days on O2	1.03	[0.99 - 1.06]	0.14		Days on O2	1.03	[0.99 - 1.06]	0.17
rs1136450	Additive G → C	1.75	[0.53 - 5.73]	0.36	rs1136450	G/G vs C/*	1.51	[0.24 - 9.56]	0.66
(LR=0.27)	GA	0.56	[0.30 - 1.04]	0.066	(LR=0.19)	GA	0.58	[0.32 - 1.06]	0.078
	Days on O2	1.03	[1.00 - 1.07]	**0.043**		Days on O2	1.04	[1.00 - 1.07]	**0.040**
rs1136451	Additive A → G	1.79	[0.31 - 10.28]	0.51	rs1136451	A/A vs G/*	1.79	[0.31 - 10.28]	0.51
{LR=0.48)	GA	0.57	[0.31 - 1.06]	0.077	(LR=0.48)	GA	0.57	[0.31 - 1.06]	0.077
	Days on O2	1.04	[1.00 - 1.07]	**0.024**		Days on O2	1.04	[1.00 - 1.07]	**0.024**
rs1059057	Additive A → G	3.90	[0.20 - 77.56]	0.37	rs1059057	A/A vs G/*	3.90	[0.20 - 77.56]	0.37
(LR=0.28)	GA	0.63	[0.35 - 1.14]	0.13	(LR=0.28)	GA	0.63	[0.35 - 1.14]	0.13
	Days on O2	1.04	[1.01 - 1.08]	**0.015**		Days on O2	1.04	[1.01 - 1.08]	**0.015**
rs4253527	Additive C → T	2.56	[0.30 - 21.63]	0.39	rs4253527	C/C vs T/*	2.56	[0.30 - 21.63]	0.39
(LR=0.31)	GA	0.54	[0.28 - 1.04]	0.064	(LR=0.31)	GA	0.54	[0.28 - 1.04]	0.064
	Days on O2	1.03	[1.00 - 1.07]	0.052		Days on O2	1.03	[1.00 - 1.07]	0.052
SFTPA2									
rs1059046	Additive A → C	2.32	[0.65 - 8.31]	0.20	rs1059046	A/A vs C/*	2.91	[0.49 - 17.26]	0.24
(LR=0.12)	GA	0.52	[0.27 - 1.00]	**0.049**	(LR=0.16)	GA	0.53	[0.28 - 1.00]	**0.049**
	Days on O2	1.03	[1.00 - 1.07]	**0.042**		Days on O2	1.03	[1.00 - 1.07]	**0.036**
rs17886395	Additive G → C	3.33	[0.70 - 15.75]	0.13	rs17886395	G/G vs C/*	6.53	[0.87 - 49.24]	0.069
(LR=0.070)	GA	0.67	[0.32 - 1.43]	0.30	(LR=0.027)	GA	0.68	[0.31 - 1.49]	0.33
	Days on O2	1.06	[1.01 - 1.11]	**0.022**		Days on O2	1.06	[1.01 - 1.11]	**0.017**
rs1965707	Additive C → T	9.10	[1.23 - 67.15]	**0.030**	rs1965707	C/C vs T/*	9.80	[1.28 - 75.13]	**0.028**
(LR=0.0070)	GA	0.45	[0.22 - 0.90]	**0.023**	(LR=0.0085)	GA	0.44	[0.22 - 0.89]	**0.022**
	Days on O2	1.03	[0.99 - 1.06]	0.13		Days on O2	1.03	[0.99 - 1.06]	0.12
rs1965708	Additive C → A	2.88	[0.55 - 15.04]	0.21	rs1965708	C/C vs A/*	2.90	[0.53 - 15.93]	0.22
(LR=0.13)	GA	0.6	[0.35 - 1.04]	0.071	(LR=0.15)	GA	0.60	[0.35 - 1.04]	0.068
	Days on O2	1.04	[1.00 - 1.07]	**0.028**		Days on O2	1.04	[1.00 - 1.07]	**0.027**
SFTPD									
rs721917	Additive T → C	0.47	[0.11 - 1.93]	0.30	rs721917	T/T vs C/*	0.17	[0.01 - 2.43]	0.19
(LR=0.20)	GA	0.52	[0.27 - 1.01]	0.055	(LR=0.80)	GA	0.53	[0.28 - 0.99]	**0.048**
	Days on O2	1.04	[1.00 - 1.07]	**0.027**		Days on O2	1.04	[1.01 - 1.08]	**0.012**
rs2243639	Additive G → A	1.72	[0.54 - 5.51]	0.36	rs2243639	G/G vs A/*	2.01	[0.39 - 10.46]	0.40
(LR=0.29)	GA	0.67	[0.37 - 1.22]	0.19	(LR=0.34)	GA	0.66	[0.36 - 1.18]	0.16
	Days on O2	1.04	[1.01 - 1.08]	**0.014**		Days on O2	1.04	[1.01 - 1.07]	**0.015**

Table S5: Risk of ROP associated with each SNP adjusted for gestational age and duration of oxygen use in infants with BPD. Each individual SNP was evaluated under each of two alternative inheritance patterns of risk: Additive (allele dosage) and Dominant. The wildtype (Wt) allele was determined as the most common allele in infants that did not have ROP. “p value” is from a Wald’s test whether OR is significantly different from OR = 1, the p value indicated by “LR =” is from a Likelihood Ratio test whether adding the haplotype to the risk model for ROP offers a significant improvement in goodness-of-fit. “p value” ≤ 0.05 are considered to be significant and highlighted in bold font. These are “adjusted” results, i.e., both GA and DO_2 are included in the risk model; the Likelihood Ratio tests compare ROP = GA + DO_2 vs ROP = SNP + GA + DO_2; Wald tests are from the latter model.

	Model terms	OR	95% CI	p value		Model terms	OR	95% CI	p value
SFTPA1									
rs1059047	T/T (n=6)				rs1059047	C/* (n=27)			
	GA	NA				GA	0.76	[0.37 - 1.56]	0.46
	Days on O2	NA				Days on O2	1.04	[0.99 - 1.10]	0.081
rs1136450	G/G (n=13)				rs1136450	C/* (n=27)			
	GA	0.55	[0.21 - 1.42]	0.21		GA	0.58	[0.24 - 1.39]	0.22
	Days on O2	1.00	[0.95 - 1.05]	0.86		Days on O2	1.08	[1.01 - 1.17]	**0.028**
rs1136451	A/A (n=26)				rs1136451	G/* (n=16)			
	GA	0.62	[0.31 - 1.23]	0.17		GA	0.51	[0.15 - 1.74]	0.28
	Days on O2	1.03	[0.99 - 1.06]	0.18		Days on O2	1.07	[0.98 - 1.17]	0.12
rs1059057	A/A (n=37)				rs1059057	G/* (n=4)			
	GA	0.70	[0.38 - 1.26]	0.23		GA	NA		
	Days on O2	1.04	[1.01 - 1.07]	**0.022**		Days on O2	NA		
rs4253527	C/C (n=33)				rs4253527	T/* (n=9)			
	GA	0.44	[0.20 - 0.93]	**0.031**		GA	NA		
	Days on O2	1.02	[0.98 - 1.06]	0.28		Days on O2	NA		
SFTPA2									
rs1059046	A/A (n=14)				rs1059046	C/* (n=28)			
	GA	0.39	[0.10 - 1.57]	0.19		GA	0.62	[0.29 - 1.29]	0.20
	Days on O2	1.03	[0.98 - 1.07]	0.25		Days on O2	1.05	[1.00 - 1.10]	0.075
rs17886395	G/G (n=24)				rs17886395	C/* (n=17)			
	GA	0.95	[0.39 - 2.34]	0.92		GA	0.12	[0.00 - 65.29]	0.51
	Days on O2	1.05	[0.99 - 1.12]	0.087		Days on O2	1.18	[0.85 - 1.64]	0.33
rs1965707	C/C (n=22)				rs1965707	T/* (n=20)			
	GA	0.07	[0.01 - 0.85]	**0.037**		GA	0.77	[0.37 - 1.62]	0.49
	Days on O2	1.03	[0.96 - 1.10]	0.46		Days on O2	1.05	[0.99 - 1.11]	0.13
rs1965708	C/C (n=28)				rs1965708	A/* (n=14)			
	GA	0.53	[0.24 - 1.18]	0.12		GA	0.59	[0.24 - 1.45]	0.25
	Days on O2	1.04	[1.00 - 1.08]	**0.028**		Days on O2	1.01	[0.94 - 1.09]	0.78
SFTPD									
rs721917	T/T (n=7)				rs721917	C/* (n=35)			
	GA	NA				GA	0.61	[0.31 - 1.18]	0.14
	Days on O2	NA				Days on O2	1.04	[1.00 - 1.07]	**0.025**
rs2243639	G/G (n=21)				rs2243639	A/* (n=21)			
	GA	0.64	[0.25 - 1.68]	0.37		GA	0.62	[0.28 - 1.39]	0.25
	Days on O2	1.08	[1.00 - 1.16]	**0.046**		Days on O2	1.02	[0.99 - 1.06]	0.23

Table S6: Comparing Effects of Gestational Age (GA) And Duration of Oxygen Treatment (Days-On-O2) On Risk of ROP In Babies with BPD Within The W/W And */M Genotype Classes for Each SNP. This aimed to show whether there was an interactive effect of the SNP on these covariates. We are looking for large changes in the covariate's OR between W/W and */m. Note: this is observational, not inferential. "p value" is from a Wald's test whether OR is significantly different from OR = 1. "p value" ≤ 0.05 are considered to be significant and highlighted in bold font. "NA": the group size was too small and the computational algorithm to fit the model failed to converge to a solution. Observationally, it appeared that n=13 is the minimum (solution reached for n=13 or more, but not for n=9 or less).
